# Scientific publication rate in disorders of consciousness research

**DOI:** 10.3389/fpsyg.2024.1389376

**Published:** 2024-06-05

**Authors:** Francesco Riganello, Walter G. Sannita

**Affiliations:** ^1^Research in Advanced Neurorehabilitation, S. Anna Institute, Crotone, Italy; ^2^Department of Neuroscience, Rehabilitation, Ophthalmology, Genetics, and Mother/Child Sciences (DINOGMI), University of Genova, Genova, Italy

**Keywords:** disorders of consciousness (DoC), neuroscience, EEG, PET, fMRI

Seminal neuroimaging reports have promoted systematic research documenting retained aspects of brain activity of varying complexity across sensory, language, emotional, or learning dynamics in subjects in vegetative state (VS) (aka *unresponsive wakefulness syndrome*; UWS) (Owen and Coleman, 2008) and minimally conscious state (MCS) (Giacino et al., 2018). As a result, the number of publications in the international literature reporting about fMRI, PET scan, and advanced-methodology EEG studies in these major disorders of consciousness (DoC) has increased steadily, according to a search on a major database (i.e., pubmed.ncbi.nlm.nih.gov). A peak was reached in yrs. 2013–2015 when the trend reversed (Figure 1), in contrast with the steady increase to date of the publication rates documented by a search in fields applying the same technologies in larger neuroscience (i.e., consciousness, neurophysiology, cognitive science, etc.), observed in the PubMed database.

**Figure 1:**
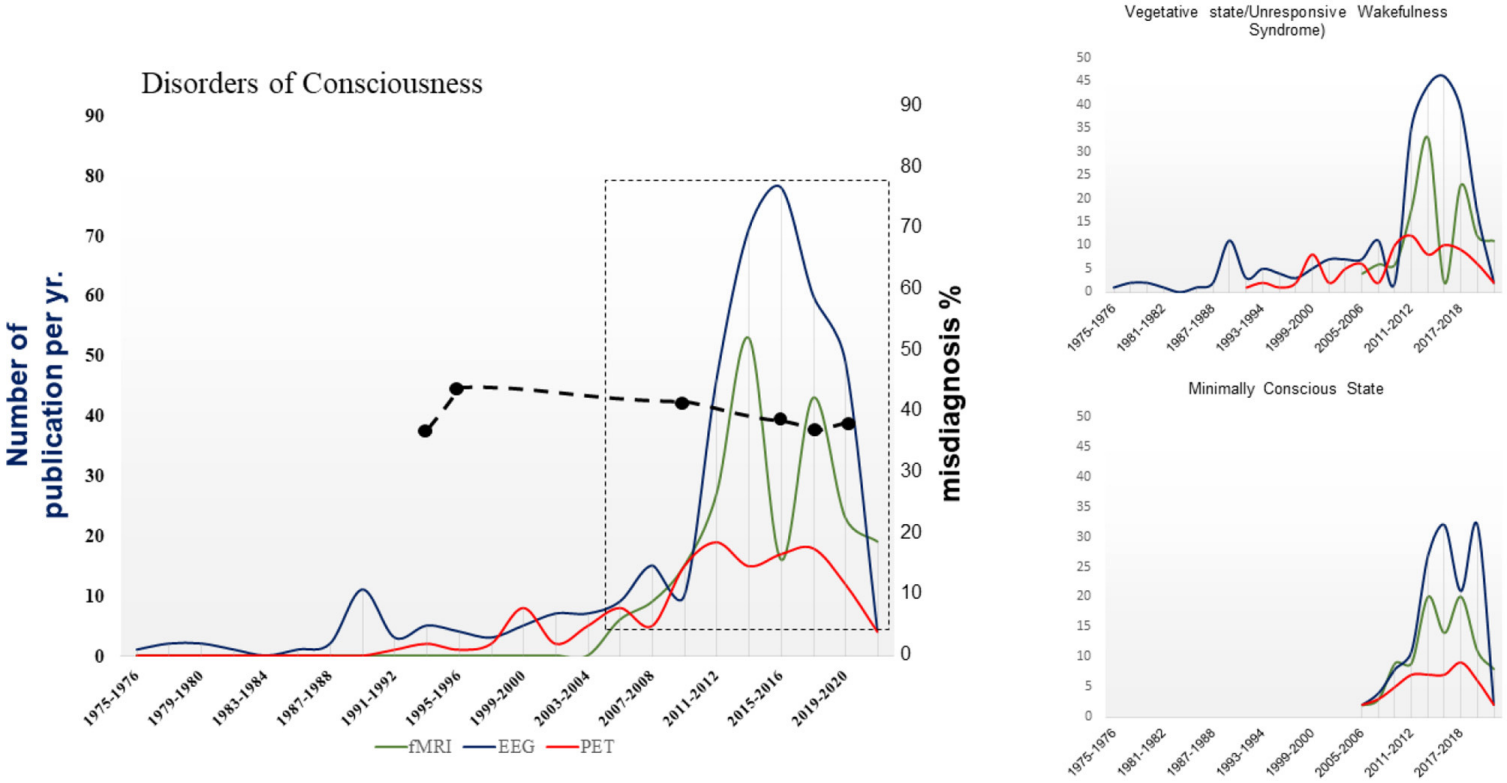
Number of papers reporting the use of fMRI, PET or electrophysiological methodologies to investigate DoC published per year in the 1975–2022 period. DoC collectively refers to the publications about vegetative state/unresponsive wakefulness syndrome and minimally conscious state due to brain damage; reviews, disorders of consciousness due to dementia, Parkinson's disease, or others are not included. Cumulative data from a bibliographic search on pubmed.ncbi.nlm.nih.gov. The publication's trend in box (from 2004 to 2022) is approximated by a quadratic curve (*p-values* of intercept, slope, and quadratic parameter = 0.0001; R^2^ = 0.62). Dashed line: percentages of estimated misdiagnosis from 1993 to 2020 (Wang et al., 2020).

The capability of the severely damaged brain to express surviving modular functions despite impaired corticocortical/cortico-subcortical connectivity has been understood as expressing retained, covert cognition/consciousness and as estimate of possible use in outcome prediction. Opponents suggested that markers of residual neural activity cannot be automatically extrapolated to qualify as surrogates for conscious activity, but only document local responsiveness of modular networks (Schiff et al., 2002; Celesia and Sannita, 2013; Farisco and Changeux, 2023; Liuzzi et al., 2023). Interest for the current diagnostic criteria and implications in medical care, legal or popular perception of bioethical issues, availability of human resources and logistics, and healthcare policies also increased, although without a steady trend.

Neuroimaging—the diffuse availability of advanced fMRI technologies in particular—has undoubtedly played a seminal role in promoting systematic research on DoC and the consequent increase in the publication rate. A nurturing effect due to some overreliance on the potentialities of neuroimaging technologies has been noted for neuroscience and is attributable to research in DoC as well (Logothetis, 2008; Snider and Edlow, 2020). The decrease of interest indicated by the contraction in the publication rate since yrs. 2013–2015, by contrast, seems to result of several factors. Effects of the constraints imposed on fMRI data interpretation by its own technology are possible (Logothetis, 2008), and are shared by PET and electrophysiology as also suggested by the parallel ongoing of publication rates over the same period of time. In particular, fMRI and PET favor the attribution of neurophysiological events to encephalic structure(s) rather than investigating time-related aspects of function(s); brain electrophysiological methods depend on frequency analyses over discrete time intervals or signal averaging over time thus reducing time discrimination. The cost-benefit ratio of extensive investigation by advanced technologies, the limited healthcare impact of new findings (with the possible exception of short-term prognosis), and the risks of misdiagnosing unchanged since 1993 (with estimated mean rates steadily around 35%) (Wang et al., 2020) should also be considered. In spite of the expanded scientific knowledge and improved technologies, consciousness seems to stand to date as a non-measurable phenomenon (Koch et al., 2016; Monti and Sannita, 2016). Direct relationships with objective neuroimaging or electrophysiological data remain difficult to interpret despite the knowledge of the brain structures and functions that are necessary for consciousness (Celesia and Sannita, 2013; Monti and Sannita, 2016). Conceivably critical in the decrease of interest in DoC research seems to have been and to be the discrepancy between the refinements in technology and research protocols and the replication of evidence that modular functions eventually surviving in DoC have limited relevance in understanding the mechanisms/functions subserving consciousness (Schiff et al., 2002; Celesia and Sannita, 2013). The issue nevertheless calls for additional research, refinement and more comprehensive application of advanced technologies reconcilable with the established neurophysiological evidence and the recent focus on theoretical modeling of consciousness and its pathophysiology (Seth and Bayne, 2022).

## Author contributions

FR: Conceptualization, Writing – original draft, Writing – review & editing, Data curation. WS: Conceptualization, Writing – original draft, Writing – review & editing.
